# Fertility intentions and child health in India: Women’s use of health services, breastfeeding, and official birth documentation following an unwanted birth

**DOI:** 10.1371/journal.pone.0259311

**Published:** 2021-11-04

**Authors:** Esha Chatterjee, Christie Sennott

**Affiliations:** 1 Department of Humanities and Social Sciences, Indian Institute of Technology Kanpur, Kanpur, Uttar Pradesh, India; 2 Department of Sociology, Purdue University, West Lafayette, IN, United States of America; University of Western Australia, AUSTRALIA

## Abstract

This study examines the relationship between women’s prospective fertility intentions and child health, measured via access to healthcare facilities for children and postpartum maternal behaviors that are indicative of future child health. We analyze two waves of nationally representative data (2005 and 2012) from the India Human Development Survey (IHDS). The analytic sample includes 3,442 non-pregnant, currently married women aged 18–40 in 2005 who participated in both rounds of the IHDS, and had at least one birth between 2005 and 2012. We investigate the influence of women’s prospective fertility intentions on access to benefits from the Integrated Child Development Services (ICDS), indicators of breastfeeding as recommended by the World Health Organization, and official documentation of births via birth certificates or registration. We find that 58 percent of births among women in the sample were labeled as unwanted. We use an adaptation of propensity score matching—the inverse-probability-weighted regression adjustment (IPWRA) estimator—and show that, after accounting for maternal and household characteristics that are known to be associated with maternal and child health, children who resulted from unwanted births were less likely to obtain any benefits or immunizations from the ICDS, to be breastfed within one hour of birth, and to have an official birth certificate. Results from this study have direct policy significance given the evidence that women’s fertility intentions can have negative implications for child health and wellbeing in the short and longer term.

## Introduction

Fertility intentions have emerged as an important determinant of maternal and child health in recent literature [1–6]. Unintended births may negatively influence women and their families by imposing financial, social, emotional, and physical costs [2, 7]. Moreover, birth intendedness is associated with maternal postpartum behaviors that are known to influence child health, such as breastfeeding. This relationship holds across regions. For example, studies from the United States have found that children resulting from unwanted births were less likely than those identified as wanted to be breastfed [8–13]. Additionally, studies from low-income countries in South America and West Africa have shown that compared to women who reported their births were wanted, women with unwanted births had lower likelihoods of breastfeeding and continuing to breastfeed [14–17]. Fertility intentions are also associated with several distinct indicators of child health across contexts, including higher rates of childhood illness [18], low birthweight [9, 19], stunting [20–22], and infant mortality [21, 23, 24].

India is an opportune setting for this study because of its high rates of unintended pregnancy and birth, and pervasiveness of poor child health. Although India was the first country to start the National Programme for Family Planning in 1952 and has continued to expand outreach and accessibility of contraceptives over the decades, considerable gaps in coverage and use remain. Only half of married women use modern contraceptives and over one in ten women (13 percent) have an unmet need for contraception [25]. The total fertility rate in India fell to around 2.2 children per woman in 2016 [26], yet estimates suggest that roughly half of pregnancies are unintended [27]. Several factors contribute to the high rate of unintendedness in India, including differences in access to contraceptives across regions, fear of side effects of modern contraception, contraceptive discontinuation, and method failure [28–31]. In terms of child health, India has the highest number of children who are stunted (about 46.6 million), accounting for 31 percent of the world’s burden for stunting [32]. In 2018, India also had the highest number of annual deaths to children under age 5 (around 882,000) [33].

Despite these trends, only a handful of studies in India have analyzed the relationship between fertility intentions and child health. Studies that have examined these relationships have shown that unintendedness is associated with several detrimental outcomes including lower rates of child vaccination [34–36] and maternal breastfeeding [37], and higher risks of child mortality [34, 35], child illness [38], preterm birth, low birthweight [37], and stunting [34, 39].

Although collectively these studies highlight the potential child health consequences of unintended births, there are several data and methodological limitations. First, most studies examining the relationship between fertility intentions and child health in India focus on one or only a few (often rural) States, which limits generalizability [see for exception 36]. Second, a majority of studies use retrospective data to measure fertility intentions [see for exception 6, 36] and research has shown that these measures may be biased, especially in underestimating unwanted births [40].

The current study builds on past research on fertility intentions and child health in India while addressing several limitations of past work. First, we analyze the first nationally representative dataset from India, which allows for a more complete and generalizable view of the association between women’s fertility intentions and their child’s health outcomes. Second, we use longitudinal data with prospective measures of fertility intentions, which allows us to mitigate the potential bias in the retrospective measures of intentions that are typically relied upon. The longitudinal data also allow us to assess how fertility intentions influence child health over time. Third, we employ a propensity score weighting approach that acts as a robustness check to help determine whether significant differences in child health are due to differential maternal or household traits or to differences in women’s fertility intentions [see 6].

This is one of the first studies in the Indian context to focus on the associations between birth intendedness and dimensions of healthcare access and postpartum behavior after accounting for women’s socio-economic characteristics. The dimensions of healthcare access and postpartum behavior studied include access to benefits from the Integrated Child Development Services (ICDS), timely initiation of breastfeeding, and birth registration. Each of these indicators has direct policy significance and could positively impact various dimensions of child health. For example, research has shown that the ICDS significantly decreases long-term child malnutrition in India [41]. Second, a multipronged strategy by the national and state governments to support breastfeeding—which is seen as a feasible and cost-effective means to improve child health and development [42]—increased early initiation of breastfeeding from 25 percent to 45 percent between 2006 and 2014 [33]. Finally, registering births and obtaining birth certificates maximizes good health and wellbeing by enabling people to access health facilities and benefits [43].

## Materials and methods

### Data and sample

We use data from two rounds of the India Human Development Survey (IHDS), which is the first nationally representative panel dataset collected in India. IHDS I conducted interviews with individuals from 41,554 households across 34 States and Union Territories in India, covering 1,503 villages and 971 urban areas [44]. In the IHDS II follow-up in 2012, 83 percent of households were re-interviewed. Questions on fertility intentions, the last birth, and child health were asked to one ever-married woman in each household in 2005. For the present study we use data from the 25,479 ever married women who took part in both rounds of the IHDS. We limited this pool of respondents to a sub-sample of 18,737 women who in the year 2005 were aged 18–40, non-pregnant, and currently married. Out of this group of women, around 9.5 percent had missing or invalid responses on the fertility intentions question and were thus dropped from the analyses. Because our key independent variable measures differences between those who had wanted and unwanted births, we included only those women who had at least one birth between 2005 and 2012 [see also 6]. Around 78 percent of women in the sub-sample were dropped because they did not have a child between 2005 and 2012. Our final sample is thus limited to 3,442 non-pregnant, currently married women aged 18–40 in 2005 who participated in both rounds of the IHDS, had at least one birth between 2005 and 2012, and had non-missing data on fertility intentions (in 2005) and other independent and control variables.

### Variables

#### Dependent variables

In the present paper we examine the relationship between women’s fertility intentions and several dependent variables that are associated with child health.

a) Benefits received from Integrated Child Development Services (ICDS): Early childhood development is key to health and wellbeing in later life [45] and therefore is an essential element of the U.N. Sustainable Development Goals, particularly goal 3 (ensure healthy lives and promote well-being for all at all ages). The ICDS were developed in 1975 to foster early childhood care and development in India. Some key services provided by the ICDS include immunization, nutrition and health education, health check-ups, preschool and non-formal education, supplementary nutrition, and referral services. The ICDS are provided through a large network known as “Anganwadi centers”. A large proportion of the immunizations that occur in rural India happen through Anganwadi centers [46, 47]. However, program design and implementation have been challenging and uneven [48–52]. For example, research has shown that the poorer Northern Indian States that have higher incidences of child malnutrition also have the lowest program coverage and budgetary allocations from the central government [51].

This is the first study to date to examine the associations between fertility intentions and benefits received from Anganwadi centers. Two items measure service receipt: i) Women were asked: “When you were pregnant and lactating with [NAME of the child], did you receive benefits from the Anganwadi center?” The question has four responses: 0 = no, 1 = while lactating, 2 = while pregnant, 3 = both. We construct a dichotomous variable that is coded 1 if the woman received any benefits from ICDS (either while pregnant, while lactating, or both), and 0 if the woman received no benefits. ii) Women were asked if the child received any immunizations from the Anganwadi center. The response is a binary variable coded 0 if the child did not receive any immunizations from ICDS and 1 if the child did receive immunizations from ICDS.

b) Breastfeeding as recommended by the WHO and UNICEF: According to the WHO—and supported by a plethora of extant research [53–55]—breastfeeding is one of the most useful means to guarantee child health and survival. Though many studies in the United States and Europe find a consistent relationship between fertility intentions and breastfeeding [2, 8, 9], one U.S. study did not find a significant relationship overall [56], and another did not find a significantly lower likelihood of breastfeeding for mistimed births [12]. Additionally, some studies have found bivariate associations between fertility intentions and breastfeeding initiation and duration, though the associations decrease significantly after including other covariates in the analyses [10, 57]. In the Indian context, only a couple of studies to date have examined the relationship between fertility intentions and breastfeeding, with mixed results. Whereas the first study was limited to the State of Andhra Pradesh and did not find a significant relationship [37], the second study used nationally representative data and found a significant relationship between birth intendedness and exclusive breastfeeding [36].

In the present study we advance past work on fertility intentions and breastfeeding in India by basing our measures of breastfeeding on the WHO and UNICEF recommendations. We constructed two dichotomous (Y/N) variables that assess whether: i) women initiated breastfeeding within the first hour of birth; and ii) women maintained exclusive breastfeeding for six months.

c) Documenting births: Wealth and regional (rural/urban) inequalities in obtaining birth certificates are prevalent in most low- and middle-income countries. Poor civil registration and vital statistics systems in South Asia and Africa in particular could lead to the systematic exclusion of children belonging to lower socio-economic statuses and rural regions from obtaining the benefits of having a birth certificate, and also prevent them from being counted in national health data [58, 59]. In India, no studies to date have examined the relationship between fertility intentions and birth registration. Though it has not been characterized as a key indicator of child health in past research, birth registration could be an important channel through which policies targeting children’s health and education may operate. In the present study we examine two dichotomous (Y/N) variables: i) whether a card was made to register the pregnancy; and ii) whether the child has an official birth certificate.

#### Independent variables

Our main independent variable measures women’s prospective fertility intentions. We evaluate if a woman’s most recent birth was wanted or unwanted by comparing the number of children a woman wanted in 2005 to the total number of children that were born between 2005 and 2012. If the total number of additional children a woman wanted in 2005 was greater than or equal to the number of children born between 2005 and 2012 (including those who died within this period), then the most recent birth was labeled as wanted [see also 6, 60]. In contrast, if the number of additional children a woman desired in 2005 was less than the number of children born between 2005 and 2012, then the last birth was labeled as unwanted. The variable measuring unwanted birth is a dichotomous variable coded 1 if the last birth was unwanted and 0 if the last birth was wanted.

We also control for several maternal and household characteristics in 2005 that have been shown to be important in past research on maternal and child health [see 6]. We include a continuous variable for women’s age (18–40) because an age-squared term was not significant. Additionally, we include variables for a woman’s number of living children in 2005; her education level (illiterate (completed no standard years of schooling), pre-primary (completed 1–4 years), primary complete (5–9), secondary complete (10–11), higher-secondary complete (12 years and some college), and college degree or higher); and a woman’s household asset quintile (five dummy variables ranging from poorest to richest quintiles).

Maternal and child health in India are also related to several other local factors. We measure women’s caste via four dichotomous variables indicating membership in a Forward Caste (FC), Scheduled Caste (SC), Scheduled Tribe (ST), and Other Backward Class (OBC) group. We also control for religion using three dichotomous variables: Hindu, Muslim, or other religion. We include an indicator of whether a woman lives in an “Empowered Action Group (EAG)” state because these states are the focus of various government health and family planning programs due to their lower education, lower status of women, less adequate administration, and higher prevalence of traditional norms and beliefs than other states. Finally, we control for whether a woman lives in an urban (1) vs. a rural (0) area.

### Analyses

Recent studies on fertility intentions and maternal and child health in the United States and India have used fixed effects models [10, 34, 61, 62] and propensity score matching [6, 9, 37] to correct for selection bias. Propensity score analyses are less sensitive to model specification errors compared to regression models [9, 63–67]. Therefore, in the present study we use an adaptation of propensity score matching (PSM)—the inverse-probability-weighted regression adjustment (IPWRA) estimator. This adjustment is useful for disentangling the impact of a woman’s fertility intentions on a child’s health outcomes from the impact of other maternal characteristics. While PSM approaches could be sensitive to bias when the treatment or the outcome model is impacted by confounding unobservable variables [68–71], IPWRA estimators are doubly robust. This means that if either one of the treatment or outcome models is specified correctly, the effects of unwanted births on child health outcomes can be consistently estimated. IPWRA estimators model both treatment and outcome models to take into account non-random assignment of the treatment [68, 72, 73]. Weighted regression coefficients are used to calculate the averages of the treatment level predicted outcomes for child health related outcomes where the weights are the inverse probabilities of treatment [73]. To model the outcomes that would relate to future child health, we use logistic regression for the binary outcome variables (received any benefits from ICDS, received any immunizations from ICDS, breastfeeding within an hour of birth, exclusive breastfeeding for 6 months, whether the child has a birth registration card, and whether the child has a birth certificate). We also account for a mother’s age, number of children in 2005, caste, religion, education level, and the region and area of residence. We use logistic regression to predict the unwantedness of a birth as a function of a similar set of socio-demographic characteristics. All data are analyzed using Stata 15 [74]. We use an alpha of 0.05 to determine statistical significance.

## Results

Table 1 shows summary statistics for the dependent and independent variables. The descriptive results show that 58 percent of births in the sample were labeled as unwanted. Table 1 also shows that around half of the mothers in the sample reported receiving any benefits from ICDS, and around 45 percent of children received any immunizations from ICDS. Half of the mothers in the sample breastfed their babies within the first hour of birth. Less than 34 percent of mothers met the WHO recommendation to exclusively breastfeed their child for 6 months. Finally, 76 percent of mothers obtained a registration card when they were pregnant with the child, whereas half obtained a birth certificate for their child. Turning to the independent variables, the largest proportions of mothers in the sample were illiterate, Hindu, belonged to Other Backward Classes, and resided in rural areas.

**Table 1:** Weighted descriptive statistics of women aged 18–40 between 2005 and 2012 (n = 3442).

		**Proportion**
**Dependent Variables** *		
Received any benefit from Anganwadi Centers/ICDS		0.511
Received any immunization from Anganwadi Centers/ICDS		0.445
Breastfeeding within one hour of birth		0.501
Exclusive breastfeeding for 6 months		0.338
Registration card obtained		0.764
Birth Certificate obtained		0.501
**Independent Variables**		**Proportion/Mean(SD)**
Unwanted Birth		0.58
Age of Mother		24.579(4.83)
Number of Children		1.766(1.258)
Mother’s Education		
	Illiterate	0.478
	Pre-primary	0.065
	Primary Complete	0.287
	Secondary Complete	0.084
	Higher Secondary Complete	0.051
	College or More	0.034
Household Asset Quintile		
	Poorest	0.229
	Second Qunitile	0.217
	Third Quintile	0.247
	Fourth Quintile	0.169
	Richest	0.138
Religion		
	Hindu	0.799
	Muslim	0.155
	Other Religion	0.046
Caste Group		
	Forward Castes	0.234
	Scheduled Caste (SC)	0.247
	Scheduled Tribes (ST)	0.075
	Other Backward Classes (OBC)	0.444
Urban		0.199
Empowered Action Group (EAG) State		0.587

* Missing values on key dependent variables are recoded as 0.

The top panel in Table 2 shows the bivariate relationship between fertility intentions and the dependent variables measuring child health outcomes with unadjusted data (i.e., predicted probabilities from bivariate logistic regressions). Bivariate results show that children resulting from unwanted births were significantly less likely to have received any benefits or immunizations from ICDS, and to have been breastfed within one hour of birth. There was no significant difference between children from wanted and unwanted births in terms of being exclusively breastfed for 6 months. Finally, mothers were significantly less likely to obtain registration cards and birth certificates for children resulting from births that were unwanted compared to births that were wanted.

**Table 2:** Analyses of key indicators of child health for wanted and unwanted births to women aged 18–40 between 2005 and 2012.

	**Wanted Birth (n = 1,404)**	**Unwanted Birth (n = 2,038)**	
**Variables**	**Unadjusted Predicted Probabilities (95% CI) (Robust SE)**	**Unadjusted Predicted Probabilities (95% CI) (Robust SE)**	**P-value**
Any benefit from Anganwadi Centers/ICDS	0.570 (CI: 0.530–0.610) (0.020)	0.469 (0.434–0.504) (0.018)	p<0.001
Any immunization from Anganwadi Centers/ICDS	0.506 (0.465–0.548) (0.021)	0.400 (0.365–0.435) (0.018)	p<0.001
Breastfeeding by one hour of birth	0.570 (0.528–0.611) (0.021)	0.452 (0.416–0.487) (0.018)	p<0.001
Exclusive breastfeeding 6 months	0.347 (0.309–0.385) (0.020)	0.331 (0.297–0.364) (0.017)	p = 0.517
Registration card obtained	0.827 (0.799–0.855) (0.014)	0.719 (0.688–0.750) (0.015)	p<0.001
Birth certificate obtained	0.622 (0.581–0.663) (0.021)	0.413 (0.378–0.448) (0.018)	p<0.001
**Variables**	**IPWRA Adjusted Predicted Probabilities (95% CI)**	**IPWRA Adjusted Predicted Probabilities (95% CI)**	**P-value**
	**(Robust SE)**	**(Robust SE)**	
Any benefit from Anganwadi Centers/ICDS	0.567 (0.521–0.613) (0.023)	0.478 (0.442–0.514) (0.019)	p = 0.003
Any immunization from Anganwadi Centers/ICDS	0.499 (0.453–0.545) (0.023)	0.401 (0.365–0.436) (0.018)	p = 0.001
Breastfeeding by one hour of birth	0.550 (0.501–0.599) (0.025)	0.466 (0.430–0.501) (0.018)	p = 0.006
Exclusive breastfeeding 6 months	0.328 (0.289–0.367) (0.020	0.325 (0.290–0.361) (0.018)	p = 0.919
Registration card obtained	0.776 (0.737–0.815) (0.020)	0.753 (0.724–0.782) (0.015)	p = 0.333
Birth certificate obtained	0.541 (0.498–0.583) (0.022)	0.470 (0.434–0.507) (0.019)	p = 0.010

In both panels we use sample weights to obtain unbiased estimates based on the population of all births at the national level. In addition, in the bottom panel, the IPWRA adjusted model accounts for the mother’s age in 2005, number of children alive in 2005, mother’s education, household wealth quintile, caste group, religious group, type of State of residence, and area of residence (urban/rural).

The bottom panel of Table 2 shows the predicted probabilities after IPWRA. These are the predicted probabilities that we would find if women with wanted and unwanted births had similar distributions of socio-demographic traits. After weighting, the significant relationships are somewhat attenuated, however, compared to children resulting from wanted births, those from unwanted births were still significantly less likely to receive any benefits or immunizations from ICDS, be breastfed within one hour of birth, and have a birth certificate.

## Discussion

Research on child health across a variety of settings has identified the intendedness of the birth to be an important determinant [1, 2, 4, 12, 18–24, 34–39]. In this study we investigated the relationship between women’s prospective fertility intentions and child health in India. Our results show that fertility intentions have a significant influence on child health, even after accounting for women’s socio-demographic traits that are likely to be associated with both fertility intentions and child health. Specifically, after employing a propensity score matching approach—the IPWRA estimator—we found that children that resulted from unwanted births in India were significantly less likely to receive any benefits or immunizations from ICDS, to be breastfed within one hour of birth, and to have an official birth certificate. Using IPWRA allows us to account for selection bias that occurs due to maternal socio-demographic traits being linked to both women’s fertility intentions and child health outcomes.

Our study builds on past work on fertility intentions and child health in several ways. First, the data we analyse come from the IHDS—the first nationally representative survey from India—which enhances the generalizability of our results. Additionally, our prospective measure of fertility intentions enables us to mitigate some of the bias found in the retrospective measures that are typically used [40, 60]. In terms of our results, although several studies have investigated the relationship between unintendedness and breastfeeding, past results have been mixed, including among studies set specifically in India [36, 37]. We extend this research by modeling our measure of breastfeeding on the WHO and UNICEF recommendations that women breastfeed within one hour of birth. Our study is also the first in the Indian context to investigate how birth intendedness is related to women’s healthcare access and postpartum health seeking behavior for the resulting child. Specifically, we examine women’s use of Integrated Child Development Services (ICDS), which research has shown is tied to a decrease in child malnutrition in India [41]. Finally, we examine the relationship between birth intendedness and mothers’ efforts to obtain a birth certificate for the resulting child. Our results indicate that women with unwanted births are less likely to seek birth certificates, highlighting one route through which longer-term poor child health outcomes may occur as these types of official documents are critical for accessing future health facilities and benefits [43].

One limitation of our study is that we are unable to disaggregate unintended births into those that are unwanted and those that are mistimed. Due to a lack of data on timing preferences, we are also unable to examine other nuances in prospective intentions, such as whether women are ambivalent or indifferent [75–77]. These limitations may have influenced our estimate of unwanted births among the sample (58 percent), which is higher than what past studies have shown. Future research employing more nuanced measures of prospective intentions among a nationally representative sample would be beneficial for developing more precise estimates of unintendedness in India.

Results from this study have direct policy significance given the evidence that women’s fertility intentions can have negative implications for child health in the short and longer term. Children resulting from unwanted births are less likely to be breastfed within an hour of birth and are less likely to receive immunizations from Anganwadi centers. These practices may result in increased risk of child illness, mortality, as well as other long-term impacts. For example, breastfeeding is associated with short-term benefits for child health [78] and survival [79, 80], as well as long-term benefits in human capital, including intelligence [81, 82]. The lack of birth certificates for children resulting from unwanted births may suggest barriers for women in accessing official pathways to gain these documents, such as government offices. Additionally, lacking such official documents might disadvantage these children later in life when it comes time to register for school or utilize other government-provided services.

Women who are facing unwanted births would benefit from several services, including increased access to high quality healthcare for themselves before, during, and after pregnancy to enable them to have healthy pregnancies and babies. Moreover, all women would benefit from increased access to modern contraception to be able to pre-emptively avoid the pregnancies they do not want. Mothers would also benefit from increased access to accurate health information, including the WHO recommendations for mothers and babies, as well as resources that allow mothers to fulfil these health targets and move India closer to meeting the U.N. Sustainable Development Goals.

## References

[1] Brown SS, Eisenberg L. Assessing program effectiveness and cost-effectiveness. In: The Best Intentions: Unintended Pregnancy and the Well-Being of Children and Families. Washington D.C.: The National Academies Press; 1995. p. 338–66. 25121228

[2] Gipson JD, Koenig MA, Hindin MJ. The effects of unintended pregnancy on infant, child, and parental health: a review of the literature. Stud Fam Plann. 2008;39(1):18–38. doi: 10.1111/j.1728-4465.2008.00148.x 18540521

[3] Singh S, Sedgh G, Hussain R. Unintended pregnancy: worldwide levels, trends, and outcomes. Stud Fam Plann. 2010;41(4):241–50. doi: 10.1111/j.1728-4465.2010.00250.x 21465725

[4] Tsui AO, McDonald-Mosley R, Burke AE. Family planning and the burden of unintended pregnancies. Epidemiol Rev. 2010;32(1):152–74. doi: 10.1093/epirev/mxq012 20570955 PMC3115338

[5] Sedgh G, Singh S, Hussain R. Intended and unintended pregnancies worldwide in 2012 and recent trends. Stud Fam Plann. 2014;45(3):301–14. doi: 10.1111/j.1728-4465.2014.00393.x 25207494 PMC4727534

[6] Chatterjee E, Sennott C. Fertility intentions and maternal health behaviour during and after pregnancy. Popul Stud (NY). 2020;74(1):55–74. 10.1080/00324728.2019.1672881 PMC6980985 31690185

[7] Smith-Greenaway E, Sennott C. Death and desirability: retrospective reporting of unintended pregnancy after a child’s death. Demography. 2016;53(3):805–34. doi: 10.1007/s13524-016-0475-9 27150965 PMC4884011

[8] Lindberg L, Maddow-Zimet I, Kost K, Lincoln A. Pregnancy intentions and maternal and child health: an analysis of longitudinal data in Oklahoma. Matern Child Health J. 2015;19(5):1987–1096. doi: 10.1007/s10995-014-1609-6 25287250 PMC4388754

[9] Kost K, Lindberg L. Pregnancy intentions, maternal behaviors, and infant health: investigating relationships with new measures and propensity score analysis. Demography. 2015;52(1):83–111. doi: 10.1007/s13524-014-0359-9 25573169 PMC4734627

[10] Joyce T, Kaestner R, Korenman S. The stability of pregnancy intentions and pregnancy-related behaviors. Matern Child Health J. 2000;4(3):171–8. doi: 10.1023/a:1009571313297 11097504

[11] Korenman S, Kaestner R, Joyce T. Consequences for infants of parental disagreement in pregnancy intention. Perspect Sex Reprod Health. 2002;34(4):198–205. 12214910

[12] Kost K, Landry DJ, Darroch JE. Predicting maternal behaviors during pregnancy: does intention status matter? Fam Plann Perspect. 1998;30(2):79–88. 9561873

[13] Taylor JS, Cabral HJ. Are women with an unintended pregnancy less likely to breastfeed? J Fam Pract. 2002;51(5):431–6. 12019050

[14] Pérez-Escamilla R, Cobas JA, Balcazar H, Holland Benin M. Specifying the antecedents of breast-feeding duration in Peru through a structural equation model. Public Health Nutr. 1999;2(4):461–7. doi: 10.1017/s1368980099000646 10656465

[15] Berra S, Rajmil L, Passamonte R, Fernandez E, Sabulsky J. Premature cessation of breastfeeding in infants: development and evaluation of a predictive model in two Argentinian cohorts: The CLACYD Study, 1993–1999. Acta Paediatr. 2001;90(5):544–51. 11430715

[16] Chinebuah B, Perez-Escamilla R. Unplanned pregnancies are associated with less likelihood of prolonged breastfeeding among primiparous women in Ghana. J Nutr. 2001;131:1247–9. doi: 10.1093/jn/131.4.1247 11285333

[17] Hromi-Fiedler A, Pérez-Escamilla R. Unintended pregnancies are associated with less likelihood of prolonged breast-feeding: an analysis of 18 Demographic and Health Surveys. Public Health Nutr. 2006;9(3):306–12. doi: 10.1079/phn2006856 16684381

[18] Jensen ER, Ahlburg DA. Family size, unwantedness, and child health and health care utilisation in Indonesia. Bull Indones Econ Stud. 2002;38(1):43–59.

[19] Eggleston E, Tsui AO, Kotelchuck M. Unintended pregnancy and low birthweight in Ecuador. Am J Public Health. 2001;91:808–10. doi: 10.2105/ajph.91.5.808 11344894 PMC1446688

[20] Marston C, Cleland J. Do unintended pregnancies carried to term lead to adverse outcomes for mother and child? an assessment in five developing countries. Popul Stud (NY). 2003;57(1):77–93. doi: 10.1080/0032472032000061749 12745811

[21] Montgomery MR, Lloyd CB, Hewett PC, Heuveline P. The consequences of imperfect fertility control for children’s survival, health, and schooling, DHS Analytical Reports No. 7. Calverton, MD; 1997.

[22] Shapiro-Mendoza C, Selwyn BJ, Smith DP, Sanderson M. Parental pregnancy intention and early childhood stunting: findings from Bolicia. Int J Epidemiol. 2005;34(2):387–96. doi: 10.1093/ije/dyh354 15561748

[23] Frenzen PD, Hogan Dennis P. The impact of class, education, and health care on infant mortality in a developing society: the case of rural Thailand. Demography. 1982;19:391–408. 7117632

[24] Chalasani S, Casterline JB, Koenig MA. Consequences of unwanted childbearing: a study of child outcomes in Bangladesh. 2007. In Annual Meeting of the Population Association of America, New York (pp. 29–31).

[25] International Institute for Population Sciences. National Family Health Survey (NFHS-3), 2005–2006: India. 2007.

[26] International Institute for Population Sciences (IIPS), ICF. National Family Health Survey (NFHS-4), 2015–2016. Mumbai, India; 2017.

[27] Singh S, Shekhar C, Acharya R, Moore AM, Stillman M, Pradhan MR, et al. The incidence of abortion and unintended pregnancy in India, 2015. Lancet Glob Heal. 2018;6:e111–120. 10.1016/S2214-109X(17)30453-9 PMC5953198 29241602

[28] Mishra US, Roy TK, Rajan I. Antenatal care and contraceptive behavior in India: some evidence from the National Family Health Survey. J Fam Welf. 1998;44(2):1–14.

[29] Roy TK, Ram F, Nangia P, Saha U, Khan N. Can Women’s Childbearing and contraceptive intentions predict contraceptive demand? findings from a longitudinal study in central India. Int Fam Plan Perspect. 2003;29(1):25–31. doi: 10.1363/ifpp.29.025.03 12709309

[30] Desai S, Wu L. Structured inequalities-factors associated with spatial disparities in maternity care in India. J Appl Econ Res. 2010;4(3):293–319. doi: 10.1177/097380101000400303 24761090 PMC3992970

[31] Jain AK, Winfrey W. Contribution of contraceptive discontinuation to unintended births in 36 developing countries. Stud Fam Plann. 2017;48(3):269–78. doi: 10.1111/sifp.12023 28398595

[32] Fanzo J, Hawkes C, Udomkesmalee E, Afshin A, Allemandi L, Assery O, et al. 2018 Global nutrition report. London, UK; 2019.

[33] UNICEF. The state of the world’s children 2019. Children, food and nutrition: growing well in a changing world. New York; 2019.

[34] Singh A, Chalasani S, Koenig MA, Mahapatra B. The consequences of unintended births for maternal and child health in India. Popul Stud (NY). 2012;66(3):223–39. doi: 10.1080/00324728.2012.697568 22783949

[35] Singh A, Singh A, Mahapatra B. The consequences of unintended pregnancy for maternal and child health in rural India: evidence from prospective data. Matern Child Health J. 2013;17:493–500. doi: 10.1007/s10995-012-1023-x 22527770

[36] Chowdhury P, Garg MK, Sk MI. Does mothers’ pregnancy intention affect their children’s preventative and curative care in India? evidence from a longitudinal survey. BMJ Open. 2021;11(4):e042615.

[37] Singh A, Upadhyay AK, Singh A, Kumar K. The association between unintended births and poor child development in India: evidence from a longitudinal study. Stud Fam Plann. 2017;48(1):55–71. doi: 10.1111/sifp.12017 28217882

[38] Jensen ER, Ahlburg DA. A multicountry analysis of the impact of unwantedness and number of children on child health and preventive and curative care. The Policy Project Washington, DC, the Futures Group. 1999.

[39] Upadhyay AK, Srivastava S. Effect of pregnancy intention, postnatal depressive symptoms and social support on early childhood stunting: findings from India. BMC Pregnancy Childbirth. 2016;16(107). doi: 10.1186/s12884-016-0909-9 27184026 PMC4867507

[40] Koenig MA, Acharya R, Singh S, Roy TK. Do current measurement approaches underestimate levels of unwanted childbearing? evidence from rural India. Popul Stud (NY). 2006;60(3):243–56. doi: 10.1080/00324720600895819 17060052

[41] Kandpal E. Beyond average treatment effects: distribution of child nutrition outcomes and program placement in India’s ICDS. World Dev. 2011;39(8):1410–21.

[42] Manikam L, Prasad A, Dharmaratnam A, Moen C, Robinson A, Light A, et al. Systematic review of infant and young child complementary feeding practices in South Asian families: the India perspective. Public Health Nutr. 2018;21(4):637–54. doi: 10.1017/S136898001700297X 29166956 PMC10260904

[43] Brolan CE, Gouda HN, AbouZahr C, Lopez AD. Beyond health: five global policy metaphors for civil registration and vital statistics. Lancet. 2017;389(10074):1084–5. doi: 10.1016/S0140-6736(17)30753-5 28322806

[44] Desai S, Dubey A, Joshi B, Sen M, Shariff A, Vanneman R (ed. Human Development in India: Challenges for a Society in Transition. New Delhi: Oxford University Press; 2010.

[45] Lu C, Black MM, Richter LM. Risk of poor development in young children in low-income and middle-income countries: an estimation and analysis at the global, regional, and country level. Lancet Glob Heal. 2016;4(12):e916–22. 10.1016/S2214-109X(16)30266-2 PMC5881401 27717632

[46] Awofeso N, Rammohan A, Iqbal K. Age-appropriate vaccination against measles and DPT-3 in India—closing the gaps. BMC Public Health. 2013;13(358). 10.1186/1471-2458-13-358 PMC3637565 23594400

[47] Rammohan A, Awofeso N. District-level variations in childhood immunizations in India: the role of socio-economic factors and health infrastructure. Soc Sci Med. 2015;145:163–72. doi: 10.1016/j.socscimed.2015.05.004 25958173

[48] Greiner T, Pyle DF. Nutrition Assessment—India. Paper presented at the World Bank-UNICEF Joint Nutrition Assessment Workshop; 2000.

[49] India G of. Child Development. Ministry of Human Resource Development, Department of Women and Child Development. New Delhi; 2000. Available from: http://wcd.nic.in/

[50] Parliament of India, Rajya Sabha (Upper House). Department-related Parliamentary Standing Committee on Human Resource Development 104th Report. 2003. Available from: http://parliamentofindia.nic.in/rs/book2/reports/HRD/Report140th.htm

[51] Lokshin M, Das Gupta M, Gragnolati M, Ivaschenko O. Improving child nutrition? The Integrated Child Development Services in India. Dev Change. 2005;36(4):613–40.

[52] Ramani KV, Mavalankar D, Joshi S, Malek I, Puvar T, Kumar H. Why should 5,000 children die in India every day? major causes of death and managerial challenges. Vikalpa. 2010;35(2):9–20.

[53] Lawrence RA. Breastfeeding: benefits, risks and alternatives. Curr Opin Obstet Gynecol. 2000;12(6):519–24. doi: 10.1097/00001703-200012000-00011 11128416

[54] León-Cava N, Lutter C, Ross J, Martin L. Quantifying the benefits of breastfeeding: a summary of the evidence. Pan American Health Organization, Washington DC. 2002 Jun;3.

[55] Binns C, Lee M, Low WY. The long-term public health benefits of breastfeeding. Asia-Pacific J Public Heal. 2016;28(1):7–14. doi: 10.1177/1010539515624964 26792873

[56] Marsiglio W, Mott FL. Does wanting to become pregnant with a first child affect subsequent maternal behaviors and infant birth weight? J Marriage Fam. 1988;50(4):1023–36.

[57] Cheng D, Schwarz EB, Douglas E, Horon I. Unintended pregnancy and associated maternal preconception, prenatal and postpartum behaviors. Contraception. 2009;79(3):194–8. doi: 10.1016/j.contraception.2008.09.009 19185672

[58] Bhatia A, Ferreira LZ, Barros AJD, Victoria CG. Who and where are the uncounted children? Inequalities in birth certificate coverage among children under five years in 94 countries using nationally representative household surveys. Int J Equity Health. 2017;16(148). doi: 10.1186/s12939-017-0635-6 28821291 PMC5562988

[59] Bhatia A, Krieger N, Beckfield J, Barros AJD, Victoria C. Are inequities decreasing? birth registration for children under five in low-income and middle-income countries, 1999–2016. BMJ Glob Heal. 2019;4(6):e001926. doi: 10.1136/bmjgh-2019-001926 31908868 PMC6936526

[60] Yeatman S, Sennott C. The sensitivity of measures of unwanted and unintended pregnancy using retrospective and prospective reporting: evidence from Malawi. Matern Child Health J. 2015;19(7):1593–600. doi: 10.1007/s10995-015-1669-2 25636647 PMC4476926

[61] Barber JS, East PL. Home and parenting resources available to siblings depending on their birth intention status. Child Dev. 2009;80(3):921–39. doi: 10.1111/j.1467-8624.2009.01306.x 19489912 PMC3070662

[62] Guzzo KB, Hayford SR. Unintended fertility and the stability of coresidential relationships. Soc Sci Res. 2012;41(5):1138–51. doi: 10.1016/j.ssresearch.2012.03.002 23017923 PMC3487158

[63] Dehejia R, Wahba S. Propensity score-matching methods for nonexperimental causal studies. Rev Econ Stat. 2002;84(1):151–61.

[64] Drake C. Effects of misspecification of the propensity score on estimators of treatment effect. Biometrics. 1993;49(4):1231–6.

[65] McCaffrey DF, Griffin BA, Almirall D, Slaughter ME, Ramchand R, Bugette LF. A tutorial on propensity score estimation for multiple treatments using generalized boosted models. Stat Med. 2013;32(19):3388–414. doi: 10.1002/sim.5753 23508673 PMC3710547

[66] Messer LC, Oakes JM, Mason S. Effects of socioeconomic and racial residential segregation on preterm birth: a cautionary tale of structural confounding. Am J Epidemiol. 2010;171(6):664–73. doi: 10.1093/aje/kwp435 20139129

[67] Stuart EA. Matching methods for causal inference: a review and a look forward. Stat Sci a Rev J Inst Math Stat. 2010;25(1):1–21. doi: 10.1214/09-STS313 20871802 PMC2943670

[68] Abadie A, Imbens GW. Large sample properties of matching estimators for average treatment effects. Econometrica. 2006;74(1):235–267.

[69] Imbens GW. Matching methods in practice: three examples. J Hum Resour. 2015;50(2):373–419.

[70] Imbens GW. Nonparametric estimation of average treatment effects under exogeneity: a review. Rev Econ Stat. 2004;86(1):4–29.

[71] Kebebe E, Shibru F. Impact of alternative livelihood interventions on household welfare: evidence from rural Ethiopia. For Policy Econ. 2017;75:67–72.

[72] Abadie A, Imbens GW. Large sample properties of matching estimators for average treatment effects. Econometrica. 2011;74(1):235–67.

[73] Cattaneo M. Efficient semiparametric estimation of multi-valued treatment effects under ignorability. J Econom. 2010;155(2):138–54.

[74] Corp Stata. Stata Statistical Software Release 15.0. College Station, TX; 2017.

[75] Sennott C, Yeatman S. Conceptualizing childbearing ambivalence: a social and dynamic perspective. J Marriage Fam. 2018;80(4):888–901. doi: 10.1111/jomf.12489 30270937 PMC6157927

[76] Miller WB, Barber JS, Gatny HH. The effects of ambivalent fertility desires on pregnancy risk in young women in the USA. Popul Stud (NY). 2013;67(1):25–38. doi: 10.1080/00324728.2012.738823 23234316 PMC3570750

[77] Miller WB, Barber JS, Schulz P. Do perceptions of their partner’s childbearing desires affect young women’s pregnancy risk? further study of ambivalence. Popul Stud (NY). 2017;71(1):101–16. 10.1080/00324728.2016.1253858 PMC5315631 27897080

[78] Binns C, Lee M. Breastfeeding and public health impacts. In: Oxford Research Encyclopedia of Global Public Health. 2019.

[79] Sankar MJ, Sinha B, Chwodhury R, Bhandari N, Taneja S, Martines J, et al. Optimal breastfeeding practices and infant and child mortality: a systematic review and meta-analysis. Acta Paediatr. 2015;104(467):3–13. doi: 10.1111/apa.13147 26249674

[80] Horta BL, Victora CG, World Health Organization. Short-term effects of breastfeeding: a systematic review on the benefits of breastfeeding on diarrhoea and pneumonia mortality. 2013.

[81] Horta BL. Breastfeeding: investing in the future. Breastfeed Med. 2019;14(S1):S-11. doi: 10.1089/bfm.2019.0032 30985202 PMC6486667

[82] Victoria CG, Horta BL, de Mola CL, Quevedo L, Pinheiro RT, Gigante DP, et al. Association between breastfeeding and intelligence, educational attainment, and income at 30 years of age: a prospective birth cohort study from Brazil. Lancet Glob Heal. 2015;3(4):E199–205. 10.1016/S2214-109X(15)70002-1 PMC4365917 25794674

